# “Developing Capabilities”. Inclusive Extracurricular Enrichment Programs to Improve the Well-Being of Gifted Adolescents

**DOI:** 10.3389/fpsyg.2021.731591

**Published:** 2021-10-11

**Authors:** Ana María Casino-García, María José Llopis-Bueno, María Gloria Gómez-Vivo, Amparo Juan-Grau, Tamar Shuali-Trachtenberg, Lucía I. Llinares-Insa

**Affiliations:** ^1^Departamento de Educación Inclusiva y Desarrollo Sociocomunitario, Universidad Católica de Valencia San Vicente Mártir, Valencia, Spain; ^2^Departamento de Didáctica General, Teoría de la Educación e Innovación Tecnológica, Universidad Católica de Valencia San Vicente Mártir, Valencia, Spain; ^3^Departamento de Psicología Social, Facultad de Psicología, Universitat de València, Valencia, Spain

**Keywords:** gifted, extracurricular enrichment, well-being, mood, school fears

## Abstract

The educational inclusion of gifted students requires not only equity but also emotional accessibility and social participation. However, different studies indicate that gifted students constitute a vulnerable group (for example, the incidence of bullying is higher). Psychosocial variables are determinants for the development and expression of giftedness, particularly during adolescence. This study analyzes the impact of an inclusive extracurricular enrichment program for gifted secondary school students on the well-being of adolescents. The program was based on the enrichment model of Renzulli and Reis (2016). The objective was to develop a cluster to facilitate high-achieving learning in collaboration with teachers, administrators, and guidance counselors from their schools as well as university professors and students that would address their emotions and socialization across the board and benefit or involve their peers in their regular classrooms. The intervention took place over two years: eight sessions, one afternoon per week, for five months during each school year. The sample consisted of 47 students from the first and second years of compulsory secondary education (*Educación Secundaria Obligatoria - ESO*) (age, mean (M) = 12.57, standard deviation (SD) = 0.82) during the first year and 27 students from the first, second, and third years of ESO (age, M = 13.48, SD = 0.94) during the second year; 61.4% were girls. Participants completed a questionnaire before (T1) and (T3) and after (T2) and (T4) each intervention. The results show better outcomes for psychological and subjective well-being, more positive moods, and a significant reduction in school fears. The results from this study indicate the importance of educational screening and support for gifted students to promote their well-being through collaborative enrichment activities.

## Introduction

### Educational Inclusion

Inclusion and equity in education are the cornerstone of the international education agenda for the coming years (UNESCO, 2016), and many countries and governments have included these items in their education policies (Ainscow, 2020; Martínez-Usarralde, 2021). Inclusive schools must ensure the highest possible development of the abilities of all pupils considering that effective education benefits all students (Ainscow, 2012, p. 40) by eliminating barriers that prevent true participation (Booth and Ainscow, 2015). In this regard, gifted students cannot be overlooked (Herranz and Sánchez, 2019). Our laws on education conform to the necessity of adequately addressing pupils’ specific characteristics, but in practice, they are not often receiving what they truly need (Ersoy and Uysal, 2018; Parr and Stevens, 2019; Rodríguez-Naveiras et al., 2019), leading to underachievement (Siegle, 2018; Lamanna et al., 2019) and even school failure and early school withdrawal (Blaas, 2014).

### Gifted Students

Who are gifted students? One of the most widely accepted theoretical models of giftedness is The Tripartite Model (Pfeiffer and Shaughnessy, 2020): “Giftedness through the lens of high intellectual ability; Giftedness through the lens of outstanding accomplishments; and Giftedness through the lens of potential to excel” (p. 376). This practical model integrates elements of earlier conceptions. In general, although many theories have been proposed, they are not mutually exclusive (Sternberg and Kaufman, 2018). At present, an evolutionary approach seems to predominate (Tourón, 2020); skills will only develop if the appropriate circumstances, educational opportunities and psychosocial variables are in place for natural abilities to be transformed into giftedness (Gagné, 2015). Depending on the baseline concept, between 5 and 15% of students are estimated to be gifted (Pfeiffer, 2017); however, the percentage of students identified in Spain is significantly lower at 0.4% (according to statistics from the Ministry of Education for the 2018-19 school year; Statistics from the Ministry of Education, 2021a, b). Teacher training is essential to detecting students’ potential (Gali et al., 2017; López et al., 2019).

As achievement depends on abilities being nurtured, activities to develop giftedness should be offered to all children as early as possible, particularly those who demonstrate interest and effort (Renzulli, 2008), primarily in the form of enrichment, both in and out of school (Subotnik et al., 2011). Underachievement is a serious problem that can frequently occur among this group (Colangelo, 2002); up to 50% will exhibit it at some point in their lives (Siegle, 2018). Underachievement is defined as the discrepancy between academic ability and outcomes (Rimm, 1997).

The percentage of Spanish students achieving at the highest levels in reading, mathematics, and science based on the Programme for International Student Assessment (PISA) is below the OECD and European Union averages (Ministerio de Educación y Formación Profesional, 2019). However, while international interest in comparing the achievement of students with potential and adopting preventive measures has increased over the last decade (e.g., US Department of Education, 2015; Thomson et al., 2016), few studies have analyzed which school-related factors may influence underachievement of the most able students (White et al., 2018).

The scientific literature points to, for example, a dearth of curricular challenges (Little, 2012); boredom can lead to a lack of motivation in gifted students, as well as poorer outcomes (Feldhusen and Kroll, 1991). An unchallenging curriculum and academic trait boredom may have a negative influence on students’ career aspirations (Krannich et al., 2019). Lower use of learning strategies may also be key (Gilar-Corbi et al., 2019). Poor family, school, and community environments may contribute to this phenomenon (Blaas, 2014; Plucker and Peters, 2018). Other problems experienced by gifted students may also lower their achievement, including maladaptive perfectionism (Yustikasari et al., 2020), social isolation (Vialle et al., 2007), stress (Suldo et al., 2009), depression, anxiety (Cross and Cross, 2015), loss of confidence (Siegle et al., 2020), twofold exceptionality (Reis and McCoach, 2002; McCoach et al., 2020), being a minority (Ford et al., 2018; Davis et al., 2020), and bullying (Bergold et al., 2019), among others.

Poor socioemotional well-being can be a factor in early withdrawal or underachievement (Blaas, 2014). Ritchotte et al. (2014) found that gifted students with low academic achievement are less emotionally engaged and more detached. Their scores for attitudes toward school and teachers are lower (McCoach and Siegle, 2003; Abu-Hamour and Al-Hmouz, 2013), although results are contradictory (Godor and Szymanski, 2017). Feeling different in an environment that is markedly anti-intellectual can lead to poor social acceptance (Cross et al., 2014; Allen, 2017), resulting in behaviors including hiding one’s ability to avoid stigmatization (Blaas, 2014; Lamanna et al., 2019). Bullying victimization also has a negative impact on academic achievement (Al-Ali and Shattnawi, 2018). However, being with peers of a similar ability and close friends decreases boredom, disruptive behaviors, depression, and anxiety and increases students’ sense of belonging (Stambaugh, 2017). Peer support has an even stronger influence on academic achievement than parental and teacher support, even when students are twice as exceptional (Wang and Neihart, 2015). Peer influence is very important for those students (Henfield et al., 2008).

Some studies have found that underachievement and disengagement increase during secondary school (Suldo et al., 2009; Barbier et al., 2019; Ireland et al., 2020). With age, problems with relationships and social acceptance by peers become more prevalent (Aperribai and Garamendi, 2020). Being able to identify those students is a crucial first step in providing a tailored response (Callahan et al., 2017) that minimizes underachievement and improves well-being (Lamanna et al., 2019).

### The Well-Being of Gifted Students

Over the past decade, education and guidance professionals (Cross, 2020), departments of education, and policymakers (Blaas, 2014) have expressed increasing concern about the well-being of gifted students. As a group, although the literature presents contradictory results (Neihart, 1999; Zeidner, 2020), they are not necessarily more likely than their peers to have disorders (Cook et al., 2020); however, when they exhibit mental health difficulties, the precipitating and impacting factors may be related to their unique characteristics (Cross and Cross, 2015). Accordingly, two exceptional learners may experience a range of emotional and social problems (Beckmann and Minnaert, 2018): frustration with school, high levels of negative emotions, adverse interpersonal relationships, etc. Some issues that also warrant consideration due to their impact on levels of well-being and increase gifted students’ vulnerability include the presence of unhealthy perfectionism (Chan, 2012), asynchronous development (Rinn and Majority, 2018), overexcitability (Guthrie, 2019), negative stereotypes associated with their condition (Aziz et al., 2021), bullying (Bergold et al., 2019), excessive pressure from the environment (Zeidner, 2017), etc. They may also need different support than their peers (Woo et al., 2017).

When considering well-being, differentiating between psychological well-being (PWB), which is measured by external criteria, and subjective well-being (SWB), which is based on the internal personal criteria of each individual, is important. For SWB, satisfaction with life (SWL, an individual’s cognitive assessment of his or her life) must be distinguished from emotional balance (the positive and negative emotions, experiences, and feelings that each person has) (Diener, 1984). Studies on psychological and subjective well-being in gifted children and adolescents are discussed from this perspective below.

With regard to psychological well-being, Kroesbergen et al. (2016) found small but significant differences among children aged 6 to 8 years in favor of students with normal or typical development; gifted students experienced lower self-concepts and social acceptance, especially the more creative students. Within the group of gifted students, levels of well-being were higher for those who were nominated by their teachers as *gifted students* and showed high academic achievement. Casino-García et al. (2021) found significantly lower scores for family, social, and physical self-concepts and for self-esteem in gifted children and adolescents aged 8 to 18 years. However, the scientific literature presents contradictory results (Hoge and Renzulli, 1993; Neihart, 1999; Litster and Roberts, 2011).

In terms of subjective well-being, especially when considering life satisfaction, research also offers different results. In a study conducted by Bergold et al. (2020), gifted adolescents showed a slight advantage in SWL, although this difference was significant only when compared to that of their normal or typical peers. Bergold et al. (2015) did not find significant differences between the SWL of identified and non-identified German students. Similarly, although no significant differences were identified in a study by Ash and Huebner (1998), school experiences had greater weight regarding SWL in the gifted group, ranking last in satisfaction in the group of non-identified students. However, Shaunessy et al. (2006) found differences in favor of the well-being of gifted students; those students did not differ in general SWL but had higher satisfaction with friendships. Notably, the subjects were part of an International Baccalaureate program. In turn, Chan (2012) differentiated subjects based on the type of perfectionism that they exhibited: healthy perfectionists were the happiest and most satisfied with their lives, and those with unhealthy perfectionism were the least happy and least satisfied with life; non-perfectionists were in between the two groups. Studies analyzing the subjective well-being of gifted children and adolescents focusing on affective balance are scarce. Vialle et al. (2007) compared identified gifted students with their peers and found that they had higher mean scores for negative emotions and lower mean scores for positive emotions; only the sadness variable was significant. Similarly, students assessed by Casino-García et al. (2019) also felt sadder than their non-identified peers and had a significantly lower emotional balance, in particular, fewer positive experiences. Bergold et al. (2020) found that gifted adolescents are slightly disadvantaged in mood states compared to their normal typically developing peers, but this difference was not significant.

Research on gifted adult subjects shows similar results. Ramiro et al. (2016) found no differences other than in their satisfaction with material well-being, with higher satisfaction in the gifted population, which they attributed to the desire for consistency between what was expected and what was achieved among the gifted. However, Vötter and Schnell (2019) found significantly lower scores for subjective well-being in gifted adults; a sense of meaningfulness was a good predictor of that well-being over time.

Some studies have focused on which factors might affect gifted students’ levels of well-being. In an analysis of socioaffective concerns based on a study conducted by Jen et al. (2016), the topics most often chosen to talk about with an adult in order of importance were emotions and feelings (stress, fear, worry, and other), future aspirations (future, university, career, etc.), relationships with other students, school, and school bullying. The need for career guidance has been underscored by some investigations (Yoo and Moon, 2006).

Notably, gifted students frequently experience bullying and cyberbullying (Allen, 2017; MacFarlane and Mina, 2018). In Spain, the figures are concerning and higher than those found for school populations of similar ages (González-Cabrera, 2018). Gifted children are more frequently cyber-victims (González-Cabrera et al., 2019). Victims and cyber-victims have worse psychological well-being characterized by more depression, stress, and anxiety, a poorer quality of life, and less social support. In fact, a quarter of those affected feel that their teacher has facilitated bullying to some extent (González-Cabrera, 2018).

A stereotype persisting among some teachers is that this group is less socially skilled, less prosocial, and less adaptable (Baudson and Preckel, 2016; López et al., 2019) and that maladjustment is associated with lower teacher motivation and perceived self-efficacy (Matheis et al., 2017). Shani-Zinovich and Zeidner (2013) stress that these students often do not view themselves as successful and do not perceive themselves to be happy people.

If students are happy and feel that they contribute to the common good, they will be more likely to thrive for their own benefit and for the benefit of society in general (Zeidner, 2020). Aspiring to develop their giftedness will be a source of personal satisfaction and self-fulfillment for students and will not harm their mental health if its cultivation is guided by teachers, family, and the community (Subotnik et al., 2011). Teachers’ awareness of their students’ talents is a predictor of psychological well-being (Kroesbergen et al., 2016). Providing gifted students with the opportunity to learn new things at school through open-ended tasks in alternative settings and in a practical manner can help them feel satisfied and enjoy their daily school lives (Gomez-Arizaga et al., 2020). The development of potential requires not only effort but also training and support (Subotnik et al., 2011).

### Programs for Gifted Students

The well-being of these students seems to depend on, among other factors, the appropriateness of the educational response (Neihart, 1999). Among the actions that can be taken with this group, enrichment is particularly noteworthy (Renzulli and Reis, 2016). “Enrichment is a term used to describe a set of programming options that extend and supplement the regular curriculum and often include topics that are not typically covered in the curriculum” (Subotnik et al., 2011, p. 23).

However, this measure is often not offered, or the activities that are proposed as enrichment are fun tasks, such as hobbies, or more extensive tasks—independent, individual projects—further differentiating them (Ireland et al., 2020). Again, a lack of teacher training seems to be key (Callahan et al., 2017; Güçyeter et al., 2017; López et al., 2019; Aperribai and Garamendi, 2020).

Gifted students need projects involving intellectual, creative, and cooperative challenges (Martin-Lobo et al., 2018). They prefer complex extracurricular subjects, authentic knowledge, real problems that require making connections between ideas, and selecting the format of the products of their learning; however, they also choose to work in teams with other students for part of the time (Kanevsky, 2011). Therefore, non-cognitive variables related to social adjustment and adaptation must also be considered in these proposals (Hernández and Gutiérrez, 2014).

Psychosocial variables are determinants in the development and expression of giftedness (Subotnik et al., 2011). Schools should identify these students and offer them activities during which they can meet other like-minded peers to reduce the stress and sense of loneliness that they sometimes feel and to address their concerns and fears, such as bullying (Vialle et al., 2007).

Most intervention programs for gifted students have focused on academic outcomes or cognitive variables (Memmert, 2006; Kuo et al., 2010; Welter et al., 2018); only a small number have focused on psychosocial aspects (Neihart, 2007). Enrichment programs can improve not only students’ academic performance (Aljughaiman, 2011; Golle et al., 2018) or motivation (Gubbels et al., 2014) but also their self-concept (Feldhusen et al., 1990; Gubbels et al., 2014), same-sex and opposite-sex relationships (Rinn, 2006), and interpersonal problem solving (Martin-Lobo et al., 2018). They have been shown to be effective in both primary (e.g., Feldhusen et al., 1990; Gubbels et al., 2014; Kim, 2016; Martin-Lobo et al., 2018) and secondary schools (e.g., Rinn, 2006; Aljughaiman, 2011; Fraleigh-Lohrfink et al., 2013; Sastre-Riba, 2013; Singh, 2013). Participants consider these programs to involve activities that enable gifted students to acquire new concepts that are not learned at school and to make friends (Sastre i Riba et al., 2015). Furthermore, the programs have generally demonstrated a positive impact on students’ socioemotional development. More studies are needed to explore the effects on specific areas at all educational levels, specifically in secondary schools (Kim, 2016). We have not been able to locate any research specifically examining the impact of enrichment on subjective well-being or school fears.

Educators, guidance counselors, and learning support staff offering more intervention programs designed to minimize the risk of poor socioemotional health is crucial (Blaas, 2014; Eren et al., 2018). These students need to be provided with opportunities where they can apply their skills in emotionally charged situations (Zeidner, 2017).

### Renzulli’s Schoolwide Enrichment Model

The term enrichment is often used in practice without a clear definition (Shaughnessy and Waggoner, 2015), although the education of gifted students should be based on scientific models (Van Tassel-Baska et al., 2006).

The SEM, which was developed in the mid-1970s in the United States and has been applied for more than 30 years, has enabled the development of effective educational programs for both gifted and talented or high-achieving students from different backgrounds (Reis and Renzulli, 2009). It aims to develop the potential and creativity of all students (Renzulli and Reis, 1994) and understands learning as a challenging and fun process. To this end, teachers must adapt the curriculum, program, and services through enrichment activities for all students. As defined by Renzulli (2008), this model consists of a set of strategies with the aim of improving, as well as performance, the student’s interest, engagement in the task and enjoyment of it. For Renzulli, the idea is not to “replace existing school structures but, rather, to apply the strategies and services that define the model to improve the structures to which schools have already made a commitment” (Renzulli, 2008, p. 37). Schools must design their own programs, adapting them to their reality, context, and students, which is what Renzulli identifies as the “continuum of services”, including learning opportunities and three services: curriculum modification, differentiation, and compacting; Type I, II, and III enrichment and enrichment clusters; and talent portfolio development.

In the clusters, students from different years, alongside teachers from different subjects and people and institutions from the community, develop a project of interest that involves high-achieving learning. They are not preplanned activities but rather require exploration and collaboration. Within or outside school hours, in sessions of approximately two hours for 6-12 weeks, problems are solved, products are created, and a service is provided, which is focused on one discipline or an interdisciplinary approach. Participants are united by an interest in the topic and by emotion. Teachers guide, facilitate, and inspire. Sessions typically end with the presentation of a tangible product or with the production of useful information to promote action, which is presented to an interested audience (Renzulli, 1997; Renzulli and Reis, 2016).

### The “Developing Capabilities (*Desarrollando Capacidades*)” Enrichment Program at the Catholic University of Valencia

Gifted students need responses within and outside the classroom that provide them with the support that they need. A wide range of resources are available for offering enrichment to students with high intellectual abilities depending on the characteristics of the educational system, the school, the students, etc. (Rodríguez-Naveiras et al., 2019), including establishing agreements with local actors associated with higher education, for example, participating in university mentorships (Ibáñez et al., 2020) or enrichment programs (Sastre-Riba, 2013). Being able to work in special programs with like-minded peers and experts is a measure valued by gifted students enrolled in mixed schools (Ireland et al., 2020). Extracurricular programs for gifted students can contribute to improving their well-being (Jen et al., 2016). The extracurricular enrichment program for gifted students at the Catholic University of Valencia (Universidad Católica de Valencia - UCV) is designed along these lines. As part of the Faculty of Teaching and Educational Sciences, the program was created with a twofold objective: (1) to provide an educational response that improves the well-being of gifted secondary school students through an extracurricular intervention program; and (2) to train teachers at the participating schools to identify these students and to participate in the process of designing a differentiated and enriched curriculum. Extracurricular enrichment thus becomes the practical part of a training course that is activated in parallel—Developing Abilities—to enable the university professors and school teachers participating in a cluster to develop the necessary skills to work with this group.

Dai et al. (2011) report a gap between what educators believe and what they actually achieve. Therefore, extracurricular enrichment programs must be evaluated for their efficacy and their effects on both the intellectual and personal development of the participants (Blaas, 2014; Sastre i Riba et al., 2015). Accordingly, the questions and hypotheses investigated in this study based on the information presented in the introduction are as follows:

*Research Question 1.* Can the psychological well-being of gifted students be improved by their participation in an extracurricular enrichment program or a cluster that also involves their teachers, some of their classmates, and other students from other schools? Based on the review of the scientific literature presented in the introduction, the well-being of gifted students may depend on the fit of the educational response. For gifted students, the recognition of their teachers is very important. These adolescents value school experiences more highly, as well as the friendship of their peers. The program provides them with experiences of academic enjoyment adapted to their interests shared by their classmates and other peers with similar interests and with the support of their teachers; thus, our hypothesis 1 is as follows: The extracurricular enrichment program will improve the psychological well-being of gifted students.
*Research Question 2.* Will participating in a cluster also improve gifted adolescents’ subjective well-being, SWL, and balance of positive and negative emotions? Will their participation improve their mood? Will they feel happier and less sad? Studies show that these students sometimes have fewer positive experiences and feel different, sadder, and lonelier. The program allows them to learn about issues of their interest in a playful way with professional experts and to participate in interesting and enjoyable experiences in a team with other teenagers with whom they can identify and share their enjoyment, leading us to formulate hypothesis 2: Participation in the extracurricular enrichment program will improve the subjective well-being of gifted students; their SWL and emotional balance will improve; and they will have a more positive mood and feel less sadness.
*Research Question 3*. Will students’ concerns and fears (in particular, fear of bullying) be significantly reduced following their participation in the program? As described in the introduction, meeting other like-minded peers can reduce the stress and sense of loneliness that gifted students sometimes feel and can also help them address their concerns and fears, such as fear of bullying. Being in contact with university professors in their areas of interest can guide them in their choices of educational pathways and career aspirations, which is why we formulate hypothesis 3 as follows: Through their participation in the program, adolescents’ school fears will be significantly reduced, especially their concerns about bullying.

## Materials and Methods

### Design and Participants

To evaluate the impact of the enrichment intervention program, a quasiexperimental research design was used. Three schools participated in the program for two academic years (2017-18, 2018-19). The intervention group was assessed at four different moments to determine the impact of the enrichment program: time 1 (T1), prior to the first intervention, time 2 (T2), following the first intervention, time 3 (T3), prior to the second intervention, and time 4 (T4), following the second intervention.

During the first year, following an information session during which the research team explained the project in detail, an annual participation agreement was signed. The program’s participants were recruited directly by school guidance counselors and teachers after they received training on the concept of giftedness (Pfeiffer, 2017). Only one school had initially identified gifted students. Until the 2017-18 school year, the administration required a Psychological and Educational Report indicating an IQ equal to or higher than 130 on a standardized intelligence test for identification, which usually included the result of a creativity test and a teacher s report (Conselleria de Cultura, Educacio i Ciencia, 1999; Arocas et al., 2002). However, the scientific literature suggests that teacher-completed screening scales can help to identify other students who may have been overlooked due to their cultural and linguistic backgrounds. Accordingly, using the new gifted student screening questionnaires developed by the Regional Ministry of Education (Conselleria de Educación) (Arocas et al., 2018), teachers collectively decided which students were potential candidates for the enrichment program (irrespective of their performance). In this study, in line with the National Association for Gifted Children (2010), students who showed outstanding levels of aptitude (an exceptional ability to reason and learn) or skill (performance) in one or more areas according to their teachers were included: mathematics, art, language, sports, etc.

The teachers invited the selected students and their parents to an information meeting with the researchers at the university. After collecting information and having their questions answered, they voluntarily gave their consent (assent in the case of children over 11 years of age) and authorization for processing of their data and image collection. One family requested that their son participate in the program, a student identified at another school, and he was admitted to the program. A total of 47 adolescents in the first and second years of compulsory secondary education (Educación Secundaria Obligatoria - ESO) participated in the study (T1-T2). Of these, only 15 had officially been identified as gifted prior to their participation. The mean age of the children was 12.57 years (SD = 0.82). Two experienced serious school bullying during the program. During the second year, the procedure was repeated, and the agreement was renewed. One of the schools had initiated its own enrichment program and decided to significantly decrease its number of participants and offered the program to them; three students from other schools did not return (the reasons are unknown). Four identified children who were not enrolled in those schools were admitted at the request of their families, without the presence of their teachers. In total, 27 adolescents aged 11 to 15 years (T3-T4) were in the first, second, and third years of ESO, 19 of whom had participated in the program the previous year. The average age of the children was 13.48 years (SD = 0.94).

Among the participants, 61.4% were girls, and 38.6% were boys. In general, the students who participated in the program were successful in school; 97% of the students passed all subjects the previous year. Their marks were outstanding (A + - A, 56.8%), very good (B + - B, 34.1%), and satisfactory (C +, 9.1%). Their average scores ranged from 7 to 10 points, with 9 points being the most frequent score. In addition, 93.2% of the students participated in extracurricular activities, 86.4% of the children lived with both parents, 45.5% had two siblings, 15.9% had three siblings, 15.9% had four siblings, and some were only children. In accordance with the Declaration of Helsinki, the study protocol was approved by the Research Ethics Committee of the institution (UCV2017-2018-35).

### Program Description

The intervention program focused on offering different clusters in accordance with the model of Renzulli and Reis (2016) and was carried out over the course of two years in two periods of five months each. It began in January with the teachers and lasted from February to June with the children. Two interventions were carried out in successive years: eight weekly sessions each year for two and a half hours during one afternoon per week. During the first year, the children had the freedom to choose one of the areas offered: social-humanistic, artistic, or scientific. Guided by professors from the university and teachers from their schools, they worked in teams and created a campaign to develop civic values and improve the inclusion and health of their adolescent peers at their respective schools while consulting with their classmates and enlisting their collaboration in certain actions. The analysis was based on their needs and interests; they conducted surveys and collected information. To achieve this, a number of professionals from different fields provided them with information and tools using a range of resources and technology. The results of this project were presented at the university (to family members, faculty members, and undergraduate students) and at the three schools that signed the agreement (to their classmates, who were the targets of the campaign). All the participants traveled to all the schools by bus for a day of social interaction. The project can be reviewed in more detail at the following link: https://somprojecte.com/enriquecimiento-altas-capacidades/.

During the second year, the program revolved around international days, namely, Europe Day, World Immunization Week, International Chess Day, International Day of Women and Girls in Science, and a new proposal, Day of Existential Meaning. Based on an analysis of their interests and learning styles, a specific day was recommended to them, although they were free to choose which one to participate in. After some initial awareness-raising activities, they selected their project, which they then turned into a podcast and/or video that they published on a blog that they themselves had created. All the participants chose to celebrate World Radio Day or World Day for Audiovisual Heritage and took an active part in Safer Internet Day. Students at UCV, faculty, and professionals again guided the students in their projects, which they then presented to their families, faculty, and students as a group at the university; however, they also sought out other interested audiences to whom to present their individual projects: The Very Illustrious College of Pharmacists of Valencia (Muy Ilustre Colegio de Farmacéuticos de Valencia), the attendees of a Logotherapy Conference, a local radio program, etc. Many prestigious professionals (politicians, activists, journalists, designers, researchers, artists, etc.) gave talks and were interviewed by the adolescents; a wide variety of topics were discussed, such as fourth-generation rights, the creation of the European Union and its governing bodies, Brexit, anti-vaccine movements, research in Corpuscular Physics, and the meaning of life. The activities were interspersed with moments of social interaction, shared reading, analysis of emotions, games, etc. Those leading the clusters participated in a new edition of the teacher training course on giftedness. The following video presents a description of the second intervention program: https://www.youtube.com/watch?v=tQIK_jVVNz8. To consult the materials, visit the blog: https://diasinternacionalesucv.blogspot.com/.

### Measures

The gifted students voluntarily completed a battery of questionnaires at UCV at four different moments prior to beginning the program and following the final session for each edition: the Psychologic Well-being Scale (BIEPS), the Satisfaction with Life Scale (SWLS), The Scale of Positive and Negative Experiences (SPANE), the Mood Questionnaire, and the School Fears Survey (Inventario de Miedos Escolares - IME).

To analyze psychological well-being, this study used the BIEPS by Martina and Castro (2000). Its psychometric properties can be found in Luna et al. (2020). The scale consists of 13 items assessing the results achieved with a given way of having lived (p. 45) scored using a three-point response scale (1 = I disagree; 3 = I agree). The scale collects information on four factors: autonomy, which refers to the ability to act independently (e.g., “I can accept my mistakes and try to improve”); psychosocial bonds, which refer to the quality of personal relationships that the adolescent establishes with other people (e.g., “I have friends to confide in”); projects, which refer to the adolescent’s goals and purposes in life (e.g., “I think I know what I want to do with my life”); and self-acceptance as a feeling of well-being with oneself (e.g., “I am quite content with the way I am”). Cronbach’s alpha coefficient for this scale indicated high internal consistency (Alpha = 0.68; Alpha_*T*__1_ = 0.69; Alpha_*T*__2_ = 0.75; Alpha_*T*__3_ = 0.88; Alpha_*T*__4_ = 0.60).

To analyze SWL, this study used the Spanish version of the SWLS (Diener et al., 1985) by Atienza et al. (2000). The scale consists of five items (five-point Likert scale: 1 = strongly disagree; 5 = strongly agree) addressing the person’s global evaluation of his or her life (e.g., “In most aspects, my life is as I would like it to be”). To do this, the person analyzes the most relevant aspects of his or her life, compares the experiences valued as good against the negative experiences, and compares all of them with a standard that he or she considers appropriate. Then, he or she evaluates satisfaction with his or her life. Cronbach’s alpha coefficient for this scale indicated high internal consistency (α = Alpha = 0.6; Alpha_*T*__1_ = 0.60; Alpha_*T*__2_ = 0.60; Alpha_*T*__3_ = 0.72; Alpha_*T*__4_ = 0.61).

To analyze SWB, we used the Spanish adaptation of the SPANE (Diener et al., 2009) by Cassaretto and Martínez (2017). Gifted students rated the frequency of six positive experiences (“Pleasant”) and six negative experiences (“Bad”). Scores were provided using a five-point Likert scale (from 1 = never to 5 = always). Cronbach’s alpha coefficient indicated high internal consistency (Alpha = 0.85; Alpha_*T*__1_ = 0.85; Alpha_*T*__2_ = 0.86; Alpha_*T*__3_ = 0.83; Alpha_*T*__4_ = 0.80).

To analyze mood, we used an adaptation of the Mood Questionnaire (Rieffe et al., 2004) by Górriz et al. (2013). This scale evaluates the frequency of four moods that adolescents felt in the previous four weeks: fear (e.g., “I feel frightened”), sadness (e.g., “I feel sad”), happiness (e.g., “I feel glad”), and anger (e.g., “I feel annoyed”). This scale consists of 16 items rated on a five-point scale (1 = never, 3 = often). Cronbach’s alpha coefficient for this scale was adequate (Alpha = 0.79; Alpha_*T*__1_ = 0.85; Alpha_*T*__2_ = 0.73; Alpha_*T*__3_ = 0.77; Alpha_*T*__4_ = 0.74).

To analyze school fears, we used the IME. The IME was constructed by Méndez (1988) and has three forms (see García-Fernández and Méndez, 2008 or García-Fernández et al., 2008, for a review). The items ask about school-related situations or experiences that may produce fear, discomfort, or displeasure and are scored on a five-point Likert scale (never, rarely, sometimes, often, always). It consists of 40 items distributed in six factors: fear of school failure and punishment (“Taking a written exam”), fear of physical discomfort (“Getting sick at school”), fear of social judgment (“Asking questions in class”), fear of anticipatory and separation anxiety (“Getting dressed for school”), fear of bullying (“Being hazed at school”), and fear in situations outside the classroom (“Studying in the library”). Cronbach’s alpha coefficient for each factor indicated internal consistency (Alpha = 0.89; Alpha_*T*__1_ = 0.89; Alpha_*T*__2_ = 0.86; Alpha_*T*__3_ = 0.87; Alpha_*T*__4_ = 0.92).

### Data Analysis

All data were analyzed with IBM SPSS Statistics 26. Cronbach’s alpha coefficients were computed to establish the internal consistency of the constructs (Clark and Watson, 1995). First, we analyzed the psychosocial characteristics of the gifted students participating in the program. We carried out this description because, following Dimitrov and Rumrill (2003), the design does not use a control group and therefore does not alter the environment of the gifted students and increases the external validity. For this purpose, descriptive analyses were carried out, and the results are presented as the mean (M) and standard deviation (SD).

Second, to evaluate the effect of the enrichment program, one-way analysis of variance (ANOVA) with repeated measures (the four times) and *t*-tests for paired times were used (paired samples, T1-T2, T2-T3, and T3-T4) following Mishra et al. (2019).

The use of the linear statistical method allowed us to include data from all four times analyzed and to make better use of the quasi experimental design for the two interventions and four times (Brady et al., 2015). Changes are presented as the mean difference. To compare means between T1 and T2, we analyzed the changes that had been elicited with the program, although their extension over time was very small (3 months), and the scales used for the topics analyzed suggest that although they are dynamic constructs, they are quite stable over time. A comparison between T2 and T3 was carried out to analyze the maintenance of changes or their volatility during a calendar year. If the changes were maintained, the program could be considered highly effective. Thus, this analysis is very relevant for the validity of the program. Subsequently, a T3-T4 comparison was carried out to analyze the changes generated in the second phase of the enrichment program. Moreover, the effect size was calculated using Cohen’s d and the *f*-test (ANOVA, effect size). Values of d less than 0.2 indicate a small effect size; values equal to or greater than 0.5 indicate a medium effect size; and values of 0.8 or greater indicate a high effect size. For the *f*-test ANOVA, values below 0.1 indicate a small effect size; values above 0.25 indicate a medium effect size, and values of 0.4 or above indicate a large effect size.

## Results

### Descriptive Study of the Variables Under Study

A descriptive study was carried out to analyze the mood, SWB, SWL, PWB, and school fears of gifted students. Regarding mood, gifted students were characterized by relatively high scores for happiness (minimum = 1.6, maximum = 2.4; M = 2.07, SD = 0.16) and anger (minimum = 1.75, maximum = 2.25; M = 1.97, SD = 0.23) and average scores for fear (minimum = 1, maximum = 2.4; M = 1.58, SD = 0.25) and sadness (minimum = 1, maximum = 2.5; M = 1.65, SD = 0.59). With regard to subjective well-being, they had high scores for positive experiences (minimum = 3.33, maximum = 5; M = 4.25, SD = 0.61) and medium scores for negative experiences (minimum = 1, maximum = 3.83; M = 2.08, SD = 0.65). These findings are consistent with the medium level of SWL (minimum = 3.5, maximum = 4.75; M = 4, SD = 0.56). The scores for psychological well-being were also relatively high. These students were primarily characterized by a positive perception of social relationships (minimum = 2, maximum = 3; M = 2.85, SD = 0.28); self-acceptance (minimum = 1.67, maximum = 3; M = 2.56, SD = 0.49) and autonomy (minimum = 1.75, maximum = 3; M = 2.59, SD = 0.31) were also two dimensions with high scores. The score for projects was also high but lower than those above (minimum = 1.33, maximum = 3; M = 2.57, SD = 0.41). With regard to perceived fears in the school environment, the scores for fear of school failure (minimum = 0.9, maximum = 3.7; M = 2.70, SD = 0.35) and bullying (minimum = 0.4, maximum = 3.80; M = 2.43, SD = 0.83) were particularly noteworthy. Nevertheless, the scores were average for those factors and very low for the others. Accordingly, gifted adolescents are characterized by high scores for psychological well-being and a high perception of positive experiences and happiness, although they also have a medium perception of negative experiences and moods, which may be the reason for their medium level of satisfaction. However, they have practically no school fears except for failure and bullying, which received medium scores.

### Evaluating the Impact of the Intervention

To analyze the effectiveness of the enrichment program, a related-group comparison study was conducted. Specifically, first, a repeated measures general linear model analysis was carried out for the program’s four assessment times. Subsequently, to explore differences in each of the interventions, comparisons of T1-T2 (pre-post test of the first intervention), T2-T3 (no treatment), and T3-T4 (pre-post test of the second intervention) were carried out using the repeated measures t-test. The results obtained at the four times are presented in Table 1, and those obtained for each of the interventions are presented in Table 2. Both tables show significant and non-significant differences and the estimated effect. As a general rule, most authors present only the existence of significant differences when evaluating intervention programs. Nevertheless, some authors indicate the importance of accepting the null hypothesis. However, the lack of significant differences is relevant, for example, when the effects of an enrichment program are maintained over time even after it has ended. They are also relevant when the group score for a factor is initially high and does not decrease over the course of the program (two school years) due to the effect of age and experiences at school. Moreover, in Table 3, we show the means and standard deviations for the four times.

**TABLE 1:** General linear model repeated-measures ANOVA results at four times.

	**Pillai test**	**ρ**	**Mauchly’s W**	**ρ**	**_*F*_**	**Sig**	**Greenhouse-Geisser**	**ρ**	**Huynh-Feldt**	**ρ**	***f*-test effect size**
**PWB**											
Autonomy	3.46	0.04	0.55	0.08	3.94	0.01					0.18
Relationships	4.06	0.02	0.64	0.23	2.44	0.07					0.12
Projects	2.10	0.13	0.90	0.88	2.16	0.10					0.10
Self-acceptance	0.76	0.53	0.92	0.93	0.73	0.53					0.03
**SWB**											
SWL	2.20	0.12	0.55	0.08	1.13	0.34					0.05
Negative experiences	11.53	0.00	0.62	0.16	6.55	0.00					0.26
Positive experiences	0.23	0.86	0.74	0.45	0.24	0.86					0.04
**MOOD**											
Happiness	3.09	0.05	0.58	0.14	2.55	0.06					0.13
Anger	2.55	0.09	0.89	0.86	2.46	0.07					0.12
Sadness	4.92	0.01	0.29	0.00	3.55	0.02					0.17
Fear	4.68	0.01	0.76	0.51	5.24	0.00					0.24
**SCHOOL FEARS OF…**											
Physical discomfort	4.04	0.02	0.48	0.03	3.33	0.02	3.33	0.04	3.33	0.03	0.15
Failure	1.70	0.20	0.58	0.13	1.23	0.30					0.06
Social evaluation	0.39	0.75	0.63	0.18	0.31	0.81					0.01
Bullying	1.74	0.19	0.46	0.02	0.58	0.62	0.58	0.57	0.58	0.59	0.03
Anxiety	2.05	0.14	0.21	0.00	0.46	0.70	0.46	0.59	0.46	0.60	0.05
External situations	1.26	0.31	0.55	0.08	1.74	0.16	1.74	0.18	1.74	0.17	0.08

**TABLE 2:** Intervention effects at four times before and after the two interventions.

	**T1-T2 (Pre-post first intervention)**			**T2-T3 (No intervention)**			**T3-T4 (Pre-post second intervention)**		
	**t**	**ρ**	**Cohen’s d**	***t***	**ρ**	**Cohen’s d**	**t**	**ρ**	**Cohen’s d**
**PWB**									
Autonomy	–2.27	0.02	0.33	1.16	0.25		–1.43	0.16	
Relationships	2.02	0.04	0.30	0.56	0.57		–2.32	0.02	0.44
Projects	–1.17	0.24		1.57	0.13		–1.72	0.09	
Self-acceptance	1.04	0.30		0.64	0.52		–1.18	0.24	
**SWB**									
SWL	–3.16	0.00	0.43	0.53	0.59		–2.58	0.01	0.47
Negative experiences	3.92	0.00	0.52	–4.74	0.00	0.75	0.20	0.84	
Positive experiences	–0.88	0.38		1.10	0.28		–0.59	0.55	
**MOOD**									
Happiness	–2.07	0.04	0.30	1.57	0.13		–1.80	0.08	
Anger	2.43	0.01	0.35	–1.16	0.26		–1.23	0.23	
Sadness	2.81	0.00	0.40	–1.59	0.12		–0.46	0.64	
Fear	2.04	0.04	0.3	–2.55	0.01	0.5	–0.66	0.51	
**SCHOOL FEARS OF…**									
Physical discomfort	3.58	0.00	0.48	–0.76	0.45		0.94	0.35	
Failure	0.97	0.33		–1.33	0.19		3.33	0.00	0.56
Social evaluation	0.29	0.77		–0.22	0.82		2.25	0.03	0.42
Bullying	1.54	0.13		–1.20	0.24		2.24	0.03	0.42
Anxiety	0.51	0.61		–0.50	0.61		1.87	0.07	
External situations	0.33	0.74		–1.68	0.10		1.90	0.06	

**TABLE 3:** The means and standard deviations at the four different times.

	**T1 (*N* = 47)**		**T2 (*N* = 47)**		**T3 (*N* = 27)**		**T4 (*N* = 27)**	
	**M**	**SD**	**M**	**SD**	**M**	**SD**	**M**	**SD**
**PWB**								
Autonomy	2.59	0.31	2.76	0.34	2.71	0.38	2.80	0.31
Relationships	2.85	0.28	2.79	0.20	2.74	0.31	2.88	0.19
Projects	2.57	0.41	2.73	0.28	2.59	0.40	2.70	0.41
Self-acceptance	2.56	0.49	2.68	0.47	2.61	0.52	2.61	0.38
**SWB**								
SWL	4.00	0.56	4.12	0.55	4.06	0.55	4.18	0.48
Negative experiences	2.08	0.65	1.53	0.40	2.09	0.57	2.16	0.64
Positive experiences	4.24	0.60	4.33	0.72	4.22	0.70	4.29	0.70
**MOOD**								
Happiness	2.07	0.16	2.16	0.23	2.07	0.35	2.21	0.25
Anger	1.97	0.23	1.92	0.18	2.01	0.32	2.10	0.28
Sadness	1.65	0.59	1.30	0.43	1.53	0.30	1.67	0.34
Fear	1.58	0.25	1.33	0.19	1.47	0.16	1.51	0.26
**SCHOOL FEARS OF…**								
Physical discomfort	0.61	0.61	0.37	0.43	0.48	0.75	0.32	0.50
Failure	2.70	0.35	2.53	0.52	2.78	0.67	2.26	0.87
Social evaluation	1.19	0.95	1.17	0.79	1.20	0.74	1.06	0.85
Bullying	2.43	0.83	2.48	0.92	2.63	1.20	2.31	1.38
Anxiety	0.37	0.51	0.34	0.48	0.37	0.41	0.25	0.33
External situations	0.24	0.27	0.27	0.35	0.44	0.42	0.29	0.40

As shown in Table 1, based on the multivariate test results, the enrichment program resulted in statistically significant differences in moods, i.e., happiness, fear, and sadness. Happiness increased as an effect of the program (MT1 = 2.07, SD1 = 0.16; MT2 = 2.16, SD2 = 0.23; MT3 = 2.07, SD3 = 0.35; MT4 = 2.21, SD4 = 0.25). In turn, fear (MT1 = 1.58, SD1 = 0.25; MT2 = 1.33, SD2 = 0.19; MT3 = 1.47, SD3 = 0.16; MT4 = 1.51, SD4 = 0.26) and sadness (MT1 = 1.65, SD1 = 0.59; MT2 = 1.30, SD2 = 0.43; MT3 = 1.53, SD3 = 0.30; MT4 = 1.67, SD4 = 0.34) decreased as an effect of the enrichment program. To explore differences in terms of each of the interventions undertaken in consecutive years, time-paired repeated measures *t*-tests were carried out (T1-T2 = first intervention; T2-T3 = no intervention; T3-T4 = second intervention; and T1-T4 = pre-post test) (Table 2). During the first program (T1-T2), the dimensions with significant differences between the pretest and posttest in the general sample were happiness, anger (MT1 = 1.97; MT2 = 1.92), fear, and sadness. Specifically, the means indicate that the extracurricular enrichment program increased happiness and reduced negative moods between T1 and T2 for all participants, and this effect was maintained until T3. The only exception was fear, which increased significantly between T2 and T3 when no intervention was taking place; additionally, between T3 and T4, no significant increase was observed, indicating that the enrichment program did not significantly change the fear score. The effect on fear can be interpreted as no increase in fear or fear control. Happiness increased significantly between the pretest and the end of the second intervention. The results also show that the effects of the first enrichment program on happiness were maintained over time, i.e., between the end of the first intervention and the beginning of the second intervention, as no significant differences were observed.

With regard to subjective well-being, the results show significant differences in negative experiences (MT1 = 2.08, SD1 = 0.65; MT2 = 1.53, SD2 = 0.40; MT3 = 2.09, SD3 = 0.57; MT4 = 2.16, SD4 = 0.64), although positive experiences and SWL did not significantly increase. The results show the effectiveness of the program, as negative experiences decreased between T1 and T2. However, the results also indicate an increase in negative experiences between T2 and T3 when the intervention stopped; however, they did not increase during the second intervention. With regard to satisfaction, both interventions generated significant effects (MT1 = 4.00, SD1 = 0.56; MT2 = 4.12, SD2 = 0.55; MT3 = 4.06, SD3 = 0.55; MT4 = 4.18, SD4 = 0.48). Specifically, SWL increased during both interventions, and the changes produced during the first intervention were maintained at the beginning of the second intervention, as the differences were not significant.

With regard to psychological well-being, the results show significant differences in autonomy (MT1 = 2.59, SD1 = 0.31; MT2 = 2.76, SD2 = 0.34; MT3 = 2.71, SD3 = 0.38; MT4 = 2.80, SD4 = 0.31) and social relationships (MT1 = 2.85, SD1 = 0.28; MT2 = 2.79, SD2 = 0.20; MT3 = 2.74, SD3 = 0.31; MT4 = 2.88, SD4 = 0.19) due to implementation of the program. Table 2 shows significant differences in autonomy as a result of the first enrichment program and that the effects were maintained after the intervention ended until T3 and T4. Significant differences were also noted between the two interventions in terms of social relationships. In this case, during the first intervention, a decrease in psychological well-being resulting from social relationships was observed, which was maintained until T3. However, with the second intervention, an increase was observed.

With regard to school fears, participation in the enrichment program led to significant differences in the fear of physical discomfort (MT1 = 0.61, SD1 = 0.61; MT2 = 0.37, SD2 = 0.43; MT3 = 0.48, SD3 = 0.75; MT4 = 0.32, SD4 = 0.50) (Table 2). For the first intervention, fear of physical discomfort was statistically significant, with a decrease that was maintained between T2-T3 and no increase during T3-T4. For the second intervention, decreases in fear of failure (MT3 = 2.78, SD3 = 0.67; MT4 = 2.26, SD4 = 0.87), bullying (MT3 = 2.63, SD3 = 1.20; MT4 = 2.31, SD4 = 1.38) and social judgment (MT3 = 1.20, SD3 = 0.74; MT4 = 1.06, SD4 = 0.85) were observed.

The effect sizes (dCohen) of the different pairs of means ranged from small to large (0.30-0.75) following the parameters established by Cohen (1988). Regarding the *f*-test, the effect size ranged from small to medium (0.12-0.26).

## Discussion

This paper analyzed the impact of an extracurricular enrichment program on the well-being of gifted students. The results obtained show the effectiveness of the enrichment program. In the first course of the program in relation to psychological well-being, the autonomy of the participants improved. Subjective well-being, life satisfaction, and affective balance also improved. Negative experiences decreased, happiness increased, and negative moods (fear, anger, and sadness) were significantly reduced. Therefore, we can claim that the subjective well-being of the participants improves after the first moment of the intervention. Regarding the perception of school fears, a significant decrease in the fear of physical discomfort was observed.

During the time when no intervention was taking place, regarding psychological well-being, improvements in autonomy were maintained. Regarding subjective well-being, the level of SWL was maintained, although an increase in negative experiences was identified for the 19 students who repeated participation. The improvement in mood states was maintained except for fear, which increased significantly. However, the decrease in fear of physical discomfort was maintained.

In the second course of the program, psychological well-being derived from social relationships increases. Participants’ life satisfaction also increases. At the affective level, the program improves the happiness of participants. Therefore, we can affirm that psychological and subjective well-being improves with the enrichment program. With respect to school fears, significant decreases in the fears of failure, bullying, and social judgment were observed.

The contribution of this study is that, from a holistic view of the student, it extends the areas of analysis of the intervention to psychological and subjective well-being, moods, and school fears. The results obtained, which are in line with those of Gomez-Arizaga et al. (2020), show that learning different material in a different way and in a different context contributes to improving the well-being of gifted students. Specifically, the students’ psychological well-being increased, their sense of mastery and self-competence improved, and their perception of the quality of their personal relationships with other people improved. They also experienced an increase in their subjective well-being overall, although the results must be qualified. Their SWL increased during the first intervention (T1-T2) and again increased significantly during the second intervention (T3-T4), with the results being maintained between the two interventions (T2-T3). However, negative experiences significantly decreased only during the first intervention and increased at the end of the program. In general, happiness increased, and sadness and fear decreased.

One possible explanation for the increase in their psychological well-being, in line with Kroesbergen et al. (2016), was that teachers became aware of their students’ ability. Two of the schools did not previously have students identified as gifted, and the program helped these students to be recognized. All of them were selected at their respective schools by their teachers after the teachers had received training. Gubbels et al. (2014) also found improvements in the self-concept and motivation of participants in their enrichment program; even though they were primary school children, the students were selected by their teachers, as in our study. However, those authors did not observe any changes in well-being. The researchers attribute the lack of improvement to two specific facts: children with emotional and behavioral problems were excluded from the program, and most of the students were already participating in enrichment activities elsewhere, which was not true in our case; we did not exclude anyone. Indeed, we are aware that several students were referred for disruptive and challenging behaviors in class, which did not occur during the program. Our students had also not received any type of support prior to the study, nor were they participating in other enrichment programs. A developmental difference was also noted between the participants in the program: Gubbels et al. (2014) worked with primary school students, and we worked with secondary school students.

The fact that so many professionals from so many different fields participated in the program may have contributed to improved well-being. The students were able to listen to their experiences, ask them about their profession, work with them, and learn more about the academic world. Furthermore, students discovered the professional lives of their teachers; they learned that their teachers painted or conducted research outside of class hours, for example. At this age, they must begin to make decisions about their future studies, and the need for vocational guidance in adolescence, particularly in this group, has been identified in a number of studies (Yoo and Moon, 2006; Jen et al., 2016).

Another possible explanation is that all the activities had the objective of making the world a better place and finding meaning in life. In line with Vötter and Schnell (2019), a sense of meaningfulness can predict subjective well-being. Emotional education experts recommend including exercises that address the meaning of life and other existential concerns in interventions with gifted students, as these are topics that worry them (Turanzas et al., 2020).

Similarly, this program assigns a great deal of importance to emotions, sharing feelings, assessing feelings, talking about feelings when discussing reading passages or preparing activities, and seeing mistakes as opportunities. Along these lines, our results show that talking about how one feels can indeed contribute to improving the well-being of these students during adolescence (Jen et al., 2016).

Finally, in a meta-analysis performed by Kim (2016), the largest effect size was observed in terms of socioemotional development for summer programs combined with those during the school year. The fact that the extracurricular program was organized by the university but involved the participation of teachers, guidance counselors, and, to a certain extent, their classmates may have had a similar influence, which would support the importance of the program being integrated into the school’s educational curriculum and having the full support of the administration as well as all stakeholders in the learning community (Renzulli and Reis, 2016).

One of the key aspects to note in our study was the lasting improvement in SWL over time. Levels of SWL were maintained during the seven months when the program was not offered. Stake and Mares (2005) stress the need to follow up on enrichment programs and assess their effects after several months. The fact that a significant percentage of students returned the following year enabled an assessment at 7-8 months after the first intervention. However, such attrition may be the reason why students who returned did not improve as much between T3 and T4; the program was more effective at the beginning. Additionally, when starting from higher levels of well-being, differences are not significant.

In comparison, although negative experiences significantly decreased during the first intervention, they increased in the months between the first and second interventions and remained the same during the second intervention. Nevertheless, these negative experiences did not affect satisfaction. This result conflicts with the detailed analysis of school fears. During those two school years, the fear of physical discomfort decreased significantly. In other words, children experienced negative feelings, but their increased autonomy and strength of relationships protected them such that negative feelings did not affect their SWL, which continued to improve, or their fears. Another explanation is that during the second program, the school teachers took turns and were not present for all sessions as they had been during the first year; perhaps not having continuous support from their teachers resulted in the lack of a reduction in scores for negative experiences. However, during the second intervention, fear of failure, fear of social judgment, and fear of bullying decreased significantly. Notably, the students did not present the projects at their respective schools on this second occasion, although they faced demanding audiences in unfamiliar environments, such as conferences.

As in the program by Sastre i Riba et al. (2015), making friends, learning concepts that are not taught at school, and acquiring knowledge in a different manner are the reasons most frequently given by students to explain their satisfaction with a program.

### Theoretical and Practical Implications

At a theoretical level, our results confirm the hypothesis that the well-being of gifted students depends on the appropriateness of the educational response (Neihart, 1999). In line with Kim (2016), our study reveals the contribution of enrichment programs to the socioemotional development of gifted students, an educational level (secondary school) with little research, in specific areas, such as psychological well-being, SWL, emotional well-being, mood, or school fears. Additionally, analyses of the effects were conducted several months after the intervention, as suggested by Stake and Mares (2005).

Our results indicate that extracurricular enrichment programs in collaboration with community and higher education institutions provide an appropriate opportunity for these students (Ireland et al., 2020) and can help prevent socioemotional health problems (Blaas, 2014; Eren et al., 2018). The program also supports teacher training (Aperribai and Garamendi, 2020), contributing to their empowerment.

### Limitations and Future Research

One limitation of this study is associated with using a checklist to select participants. Furthermore, only the opinions of the teachers and guidance counselors at the schools were considered; the comments of families, peers, or the students themselves (self-nomination) were not requested. This tool was selected because it is used by the school administration to enable teachers to detect gifted students at schools and guidance counselors to initiate social, psychological, and educational assessments of those students to evaluate their possible needs. Teachers were able to collectively select students in a timely manner, and the screening scale allowed teachers—who had received only limited training—to choose students with the aforementioned characteristics. Additionally, the instruments included aspects that were not only cognitive or academic but also subjective, such as “Shows a subtle sense of humor” or “Expresses and controls their emotions.”

The assessment could have been completed with other instruments. Children who were not assessed were not given any psychometric test of intelligence or creativity nor were their school marks considered. Controlling for these variables in future studies would be desirable.

The sample was representative of the percentage of the school population with giftedness, but its size was small compared to the standard procedures for achieving statistical adequacy. As such, further research involving a larger sample of students and the participation of more schools are necessary. Training more teachers and measuring their beliefs about self-efficacy, their level of engagement in the enrichment program, and the curricular differences found in regular classes are also advisable.

In terms of the results obtained, possible interactions between the contextual and intervention-specific variables are difficult to identify. Potential threats to the validity of the information should be considered. Measuring the impact of the program on the participants’ involvement in the study and on their academic outcomes would be desirable. Improvements were identified when comparing adolescents’ scores over time; comparing these scores with those of the adolescents’ classmates who did not participate in the program likely would have been interesting.

Applying similar experiences in primary schools, particularly in the final years when the first school incidents—the first cases of bullying—begin to occur, would also be interesting.

## Conclusion

The well-being of gifted students can be improved through group educational activities selected according to their interests that involve an emotional and cognitive challenge undertaken with students of their own age and from their own school and others in their area with whom they share interests, including—in a transversal manner—activities for the development of emotional intelligence and social skills in accordance with their needs, with the support of adults, their teachers, and other social actors in the environment, such as university professors, parents, and friends. Extracurricular enrichment programs are a valid option for improving gifted students’ SWL and reducing their fears, particularly the fear of bullying. Teacher training is essential for detecting which students have potential and subsequently addressing their needs.

## Data Availability Statement

The original contributions presented in the study are included in the article/supplementary material, further inquiries can be directed to the corresponding author/s.

## Ethics Statement

The studies involving human participants were reviewed and approved by Research Ethics Committee of the institution Universidad Católica de Valencia (UCV2017-2018-35). Written informed consent to participate in this study was provided by the participants’ legal guardian/next of kin. Written informed consent was obtained from the individual(s), and minor(s)’ legal guardian/next of kin, for the publication of any potentially identifiable images or data included in this article.

## Author Contributions

All authors were responsible for the study design, data analysis, interpretation of the results, and writing of the manuscript.

## Conflict of Interest

The authors declare that the research was conducted in the absence of any commercial or financial relationships that could be construed as a potential conflict of interest.

## Publisher’s Note

All claims expressed in this article are solely those of the authors and do not necessarily represent those of their affiliated organizations, or those of the publisher, the editors and the reviewers. Any product that may be evaluated in this article, or claim that may be made by its manufacturer, is not guaranteed or endorsed by the publisher.
